# Supplementary material for: Association between Early Phase Serum Lactate Levels and Occurrence of Delayed Neuropsychiatric Sequelae in Adult Patients with Acute Carbon Monoxide Poisoning: A Systematic Review and Meta-Analysis
Source: J Pers Med. 2022 Apr 18;12(4):651. doi: 10.3390/jpm12040651 (PMC9028543; doi:10.3390/jpm12040651)
Supplement: Supplementary file 1 [file jpm-12-00651-s001.zip › jpm-1659444-supplementary.pdf]

Supplemental Table S1. Search strategy.

**Embase** (searched on 5 January 2022)		
No	Search Terms	Results
1	exp Carbon Monoxide Poisoning/ or carbon monoxide poisoning.ti,ab,kw. or carbon monoxide intoxication.ti,ab,kw. or CO poisoning.ti,ab,kw. or CO intoxication.ti,ab,kw.	6180
2	lactate.mp. or exp lactate/ or lactate.ti,ab.	251,903
3	1 and 2	239
**MEDLINE** (searched on January 5, 2022)		
No	Search Terms	Results
1	exp Carbon Monoxide Poisoning/ or carbon monoxide poisoning.ti,ab,kw. or carbon monoxide intoxication.ti,ab,kw. or CO poisoning.ti,ab,kw. or CO intoxication.ti,ab,kw.	6071
2	lactate.mp. or exp lactate/ or lactate.ti,ab.	149,951
3	1 and 2	108
**KoreaMed** (searched on January 5, 2022)		
No	Search Terms	Results
1	(("carbon monoxide"[ALL])) AND ("lactate"[ALL])	6
**KMBASE** (searched on January 5, 2022)		
No	Search Terms	Results
1	([ALL=carbon monoxide] AND [ALL=lactate])	3
**KISS** (searched on January 5, 2022)		
No	Search Terms	Results
1	ALL = carbon monoxide AND ALL = lactate	7
**NDSL** (searched on January 5, 2022)		
No	Search Terms	Results
1	ALL =carbon monoxide AND ALL =lactate	16
**KISTi** (searched on January 5, 2022)		
No	Search Terms	Results
1	(BI: CARBON MONOXIDE) AND (BI: LACTATE)	90
**RISS** (searched on January 5, 2022)		
No	Search Terms	Results
1	ALL: carbon monoxide <AND> ALL: lactate	17

Supplemental Table S2. Baseline characteristics of patients included in the meta-analysis for the association between initial serum biomarker and delayed neuropsychiatric sequelae in carbon monoxide poisoning patients.

**Study**	**Number of Pts. DNS/Total (%)**	**Age, year**	**Male, *N* (%)**	**HBOT, *N* (%)**
Han 2021 [19]	38/203 (18.7)	DNS/non-DNS, 43 (32-54) / 44 (34-57)	136 (67.0)	203 (100)
Jeon 2018 [20]	101/387 (26.1)	42.0 (32.0–56.0)	244 (63.0)	356 (92.0)
Kim Y 2018 [21]	10/102 (9.8)	55.5 (36.8–69)	59 (57.8)	97 (95.1)
Kokulu 2020 [22]	54/183 (29.5)	38.0 (28.0–53.0)	110 (60.1)	116 (63.4)
Lee 2021 [24]	12/126 (9.5)	36 (26–52)	75 (54.3)	129 (93.5)
Nah 2020 [18]	30/154 (19.5)	DNS/non-DNS, 40 (29-50) / 44 (35-55)	101 (65.6)	154 (100)
Park 2012 [23]	10/71 (14.1)	34.4 ± 14.1	51 (71.8)	15 (21.1)
Pepe 2011 [17]	34/141 (24.1)	42 ± 21	64 (45.3)	55 (39.0)

Continuous variables are presented as mean ± SD or median (interquartile range). Abbreviations: CO, carbon monoxide; DNS, delayed neuropsychiatric sequelae; HBOT, hyperbaric oxygen therapy; Pts., patients.

Supplemental Table S3. Definition of delayed neuropsychiatric sequelae.

Study	Definition and/or Assessment of DNS
Han 2021 [19]	DNS was defined as any neurological symptom or sign that newly developed within 3 months of discharge from the hospital; these could include motor deficits, cognitive decline, dysphagia, dysarthria, dyspraxia, Parkinsonism, seizures, psychosis and depression.
Jeon 2018 [20]	DNS was defined as any neurological symptom or sign that newly developed within 6 weeks of discharge from the emergency department; these could include motor deficits, cognitive decline, dysphagia, dysarthria, dyspraxia, Parkinsonism, seizures, psychosis and mood disorders. We evaluated DNS as follows. First, neurology consultations were routinely requested for the assessment of neurological signs before discharge. Second, patients were informed of DNS symptoms and our contact information. Third, patients were invited to regular follow-up visits in the neurology clinic after discharge. Fourth, we performed a telephone interview with either the patient or a surrogate using a structured questionnaire to evaluate DNS. Fifth, neurologists evaluated DNS, but objective tools for documenting DNS were not used as dedicated tools to specify DNS were not available.
KimY 2018 [21]	Discharged patients were followed for at least 2 months based on Choi's observation that the lucid interval is generally from 2 to 40 days. The presence of the following symptoms and signs, including mental deterioration, cognitive dysfunction, amnesia, gait disturbance, mutism, urinary or fecal incontinence, psychosis, depression and Parkinsonism, were assessed.
Kokulu 2020 [22]	DNS was defined as any new neurological symptoms or signs, including dysarthria, dysphagia, dyspraxia, cognitive decline, Parkinsonism, motor deficits, seizures, psychosis and mood disorders, which developed within 6 weeks of discharge from the ED.
Lee 2021 [24]	In this study, we diagnosed patients who presented with disorientation or cognitive impairment within 6 weeks of acute exposure with a high intensity of brain white matter on brain MRI, which is characteristic of DNS. Disorientation and cognitive impairment were measured using the Mini-Mental State Examination (MMSE). The MMSE was measured by the emergency medicine physicians who are trained to administer this test.
Nah 2020 [18]	DNS were defined as neurological abnormalities that occurred within 3 months of discharge and comprised symptoms such as insomnia, headache, dizziness, Parkinson-like syndrome, movement disorder, conscious disorder, mood disorder and memory disorder.
Park 2012 [23]	We defined DNS as the delayed onset of Parkinsonism, psychosis, cognitive dysfunction, memory impairment, difficulty in communication, difficulty in walking or urinary incontinence.
Pepe 2011 [17]	DNS were defined as the presence of neurological, cognitive or affective disorders, developed after hospital discharge. Neuroradiological imaging (head CT/head MRI or SPECT) was proposed to patients suffering from DNS.

Abbreviations: CO, carbon monoxide; CT, computed tomography; DEACMP, delayed encephalopathy after acute carbon monoxide poisoning; DNS, delayed neuropsychiatric sequelae; ED, emergency department; MMSE, Mini-Mental State Examination; MRI, magnetic resonance imaging; NS, neuropsychiatric sequalae; SPECT, single photon emission computed tomography.

Supplemental Table S4. Summary of subgroup analysis.

**Characteristics**	**Lactate**						
	**N**	**SMD**	**95% CI**	***p*-Value for heterogeneity**	**I², %**	**Subgroup Differences**	
						***p*-Value**	**I², %**
All studies	8	0.31	0.11–0.50	0.08	44		
Hyperbaric oxygen therapy						0.07	68.8
All patients (100%)	2	0.06	[-0.27]–0.39	0.21	36		
Partial patients (<100%)	6	0.40	0.22-0.58	0.08	44		
Nation						0.81	0
Korea	6	0.33	0.07–0.58	0.07	51		
Other nations	2	0.27	[-0.15] –0.69	0.13	56		
Quality of study						0.96	0
High quality study	3	0.31	[-0.14] –0.76	0.02	74		
Low quality study	5	0.32	0.12–0.53	0.31	17		
Sample size						0.51	0
Large sample size (≥150)	4	0.26	0.04–0.48	0.10	52		
Small sample size (<150)	4	0.42	0.00–0.85	0.12	49		

Abbreviations: CI, confidence interval; DNS, delayed neuropsychiatric sequelae; ED, emergency department; HBOT, hyperbaric oxygen therapy; N, number; SMD, standardized mean difference.

Supplemental Table S5. Summary of standardized mean difference for the association between early phase lactate and occurrence of delayed neuropsychiatric sequelae in acute carbon monoxide poisoning patients with sensitivity analysis.

Study	SMD	95% CI	*p* Value for Heterogeneity	I^2, %
Omitting Han 2021 [19]	0.38	0.23–0.53	0.37	8
Omitting Jeon 2018 [20]	0.29	0.05–0.54	0.06	50
Omitting Kim Y 2018 [21]	0.28	0.08–0.47	0.10	44
Omitting Lee 2021 [24]	0.27	0.08–0.45	0.14	37
Omitting Kokulu 2020 [22]	0.28	0.05–0.51	0.07	48
Omitting Nah 2020 [18]	0.32	0.09–0.54	0.05	52
Omitting Park 2012 [23]	0.32	0.10–0.53	0.05	52
Omitting Pepe 2011 [17]	0.34	0.14–0.54	0.09	45

Abbreviations: CI, confidence interval; SMD, standardized mean difference.

Supplemental Table S6. Evidence profile of main results using GRADE.

Result	Quality assessment							
	Participants (studies)	Design	Risk of bias	Inconsistency	Indirectness	Imprecision	Publication bias	Overall quality of evidence
Lactate for predicting DNS	1350 (8)	Observational studies	No serious	No serious	No serious	No serious	Undetected	Low
Result	Summary of findings							Importance
	No of patients		Effect					
	DNS	Non-DNS	Relative	Absolute				
Lactate for predicting DNS	305	1045		SMD 0.31 (0.11–0.50)				Critical

Abbreviations: DNS, delayed neuropsychiatric sequelae; GRADE, the Grading of Recommendations Assessment, Development and Evaluation; SMD, standardized mean difference.

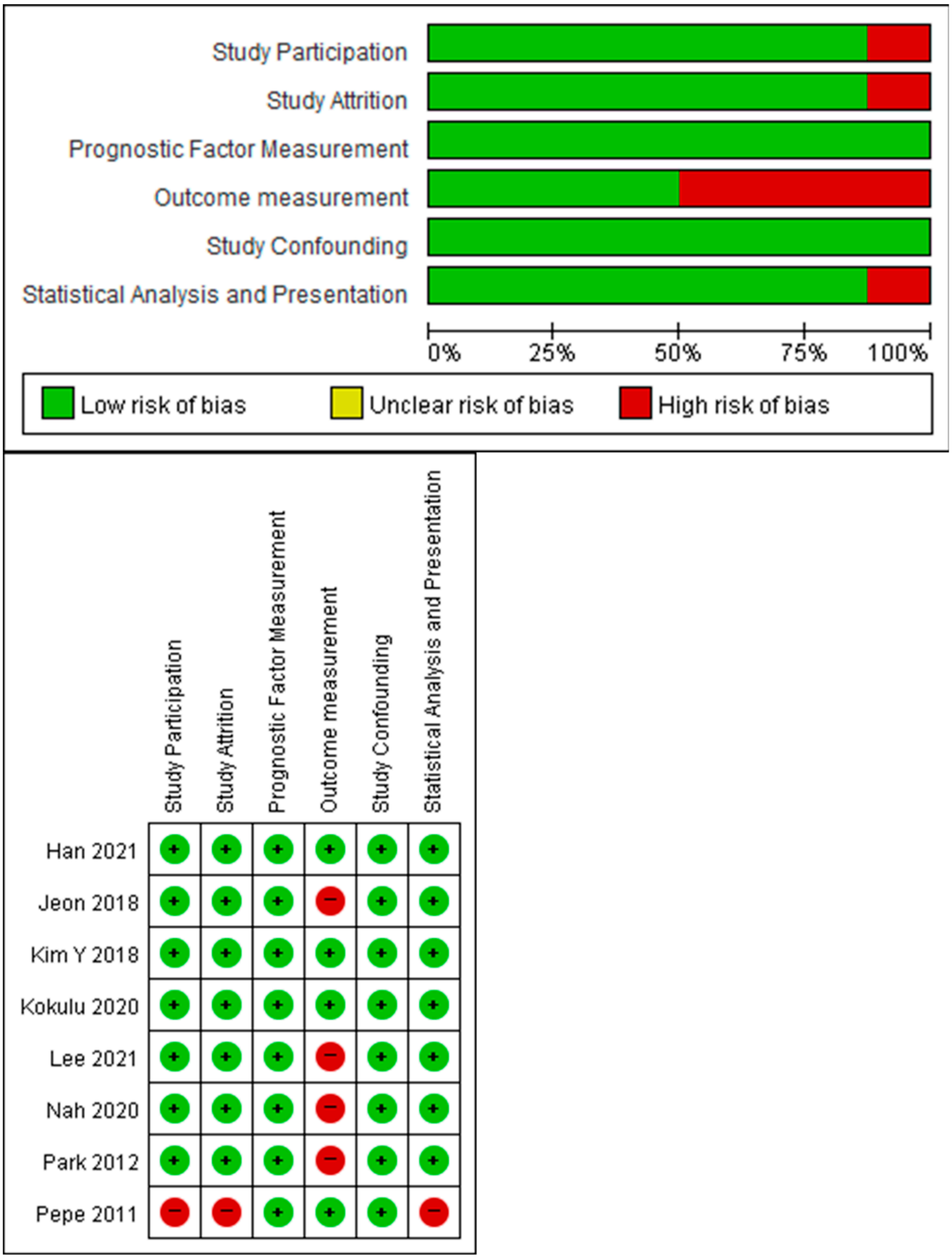

Supplemental Figure S1. Quality of the studies included in the systematic review and meta-analysis [17-24].

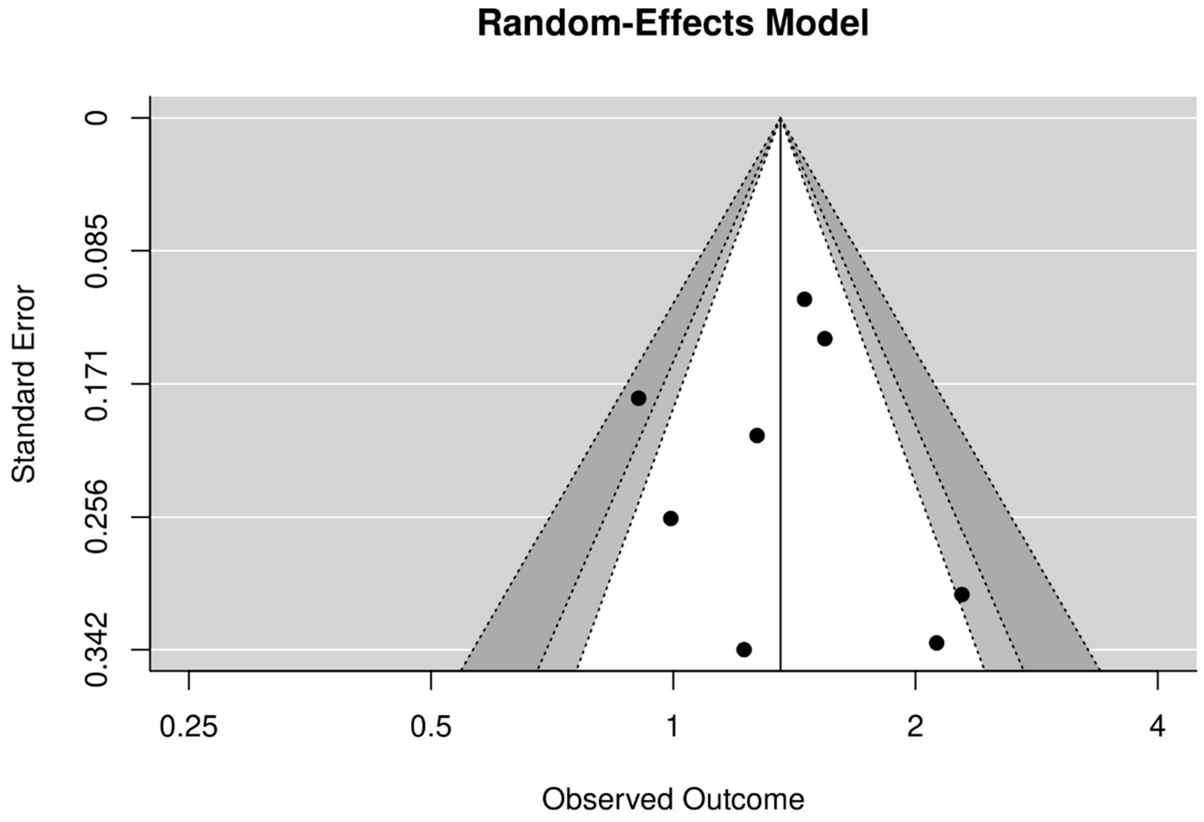

Supplemental Figure S2. Funnel plot to assess the publication bias for the association between early phase serum lactate level and occurrence of delayed neuropsychiatric sequelae in acute carbon monoxide poisoning patients.

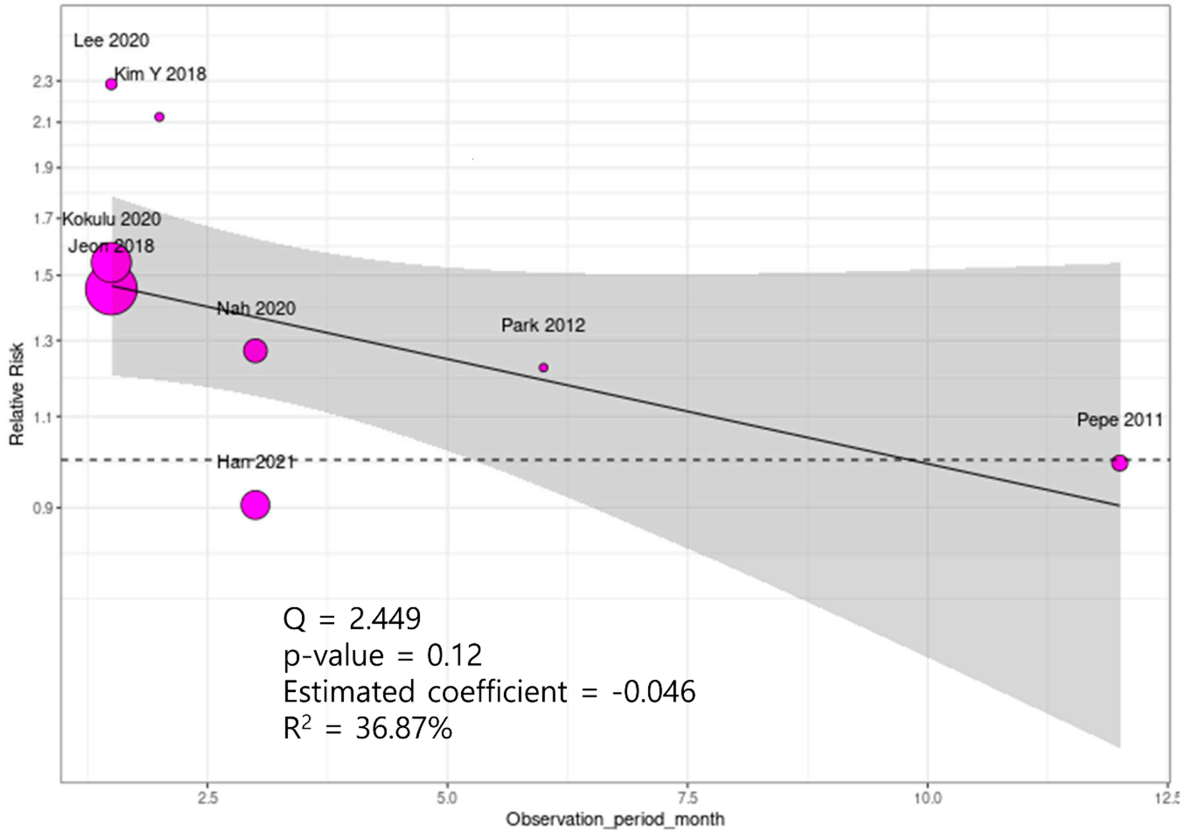

Supplemental Figure S3. Mixed-effects model meta-regression analysis: Association between the initial lactate level and occurrence of delayed neuropsychiatric sequelae in acute carbon monoxide poisoning patients determined using observation period regression [17-24].
